# Phenotype and genotype of carbapenem-resistant hypervirulent Klebsiella pneumoniae in a teaching hospital in Shanghai, China

**DOI:** 10.1099/jmm.0.001960

**Published:** 2025-03-05

**Authors:** Wei Ma, Yuxiang Wan, Xuejiao Li, Xiaochun Huang, Changzi Deng, Qin Qin

**Affiliations:** 1 Department of Laboratory Diagnostics, Shanghai Changhai Hospital, The First Affiliated Hospital of Second Military Medical University, Shanghai 200433, PR China; 2 Department of Laboratory Medicine, The International Peace Maternity and Child Health Hospital, Shanghai Jiao Tong University, Shanghai 200433, PR China

**Keywords:** carbapenem-resistant hypervirulent *Klebsiella pneumoniae* (CR-hvKP), multidrug-resistant, sequence type 11 (ST11), virulence plasmid

## Abstract

**Introduction.** Carbapenem-resistant hypervirulent *Klebsiella pneumoniae* (CR-hvKP) is an emerging pathogen associated with severe clinical outcomes, prompting an urgent investigation into its genomic characteristics and pathogenic potential.

**Hypothesis/Gap Statement.** We hypothesize that CR-hvKP strains exhibit high-level resistance and high virulence, leading to their rapid spread in clinical settings and posing a serious threat to clinical treatment.

**Aim.** The aim of the study was to investigate the phenotype and genotype of CR-hvKP strains, reveal their resistance- and virulence-related genomic characteristics and elucidate the biological characteristics of high-virulence and high-resistance strains to provide molecular epidemiological data for clinical use.

**Methodology.** Carbapenem-resistant *K. pneumoniae* (CRKP) strains were obtained from clinical samples, from January 2013 to December 2018. PCR amplification was conducted to screen for carbapenem genes. To evaluate the virulence potential of the isolates, we conducted various tests, including a string test, Galleria mellonella larvae infection test, capsular polysaccharide synthesis genotyping and genetic sequencing analyses. We used PFGE, multilocus sequence typing and next-generation sequencing to detect the genetic relationship and homology of the strains.

**Results.** In this study, we obtained 500 strains of CRKP, among which 18 strains were identified as CR-hvKP. All CR-hvKP strains were multidrug-resistant, exhibiting high-level resistance to most *β*-lactam antibiotics, including carbapenems. All CR-hvKP strains except N5 were positive for *bla*KPC-2, of which 14 isolates belonged to capsular serotype K64. Ten unrelated PFGE types were identified by PFGE analysis. Based on the results of PFGE, a total of 12 CR-hvKP isolates were selected from the 18 isolates for further testing, and 9 isolates had high homology with pLVPK virulence-related plasmids. All CR-hvKP strains showed high virulence in the Galleria mellonella infection model.

**Conclusions.** The study revealed the resistance- and virulence-related genomic characteristics of CR-hvKP strains and confirmed the high virulence of these strains. These results are of great significance for understanding the epidemiological characteristics and clinical treatment of CR-hvKP and provide basic data for the formulation of corresponding prevention and control strategies.

## Data availability

The datasets generated during and analysed during the current study are available in the NCBI repository: https://www.ncbi.nlm.nih.gov/datasets/genome/. Specific gene numbers were listed in the Supplementary Material.

## Introduction

*Klebsiella pneumoniae* is an opportunistic human pathogen that causes community-acquired and hospital-acquired infections in individuals with chronic comorbidities [1]. It causes a range of serious infections, such as pneumonia, intraabdominal infections, urinary tract infections and device-associated infections [2]. Due to the widespread use of antibiotics in recent years, *K. pneumoniae* has acquired a high antibiotic resistance rate. Indeed, carbapenem-resistant * K. pneumoniae* (CRKP) is present worldwide, and it often exhibits multidrug resistance or pan-drug resistance, thereby posing great challenges to public health and the clinical treatment of infectious diseases [3]. Resistance of CRKP to *β*-lactams is usually mediated by the enzyme *K. pneumoniae* carbapenemase (KPC), which is encoded by a gene that exists on mobile genetic elements, allowing the resistance genes to spread horizontally between different strains [4,5]. Approximately 60% of CRKP isolates in China are sequence type 11 (ST11), and KPC-2-type carbapenemase gene (*bla*KPC-2) is the most dominant type [6]. The carbapenemase *bla*KPC-2 gene is found on a Tn4401 transposon –the most common mobile genetic element associated with the *bla*KPC genes [7].

In addition to the increasingly severe trend of drug resistance, a new significant clade of *K. pneumoniae*, namely, hypervirulent *K. pneumoniae* (hvKP), has been detected in the clinical setting. This variant was first described in Taiwan in 1986 when a pyogenic liver abscess occurred in a patient without a history of hepatobiliary disease, and it has subsequently been found worldwide, especially in Asia, with the most common serotypes being K1 and K2 [8,9]. Unlike classic * K. pneumoniae* (cKP), hvKP is able to cause disease in healthy young individuals, leading to serious community-acquired infections such as liver abscess, pneumonia, purulent meningitis and endophthalmitis, and even primary osteomyelitis [10,11]. The mortality rate of hvKP infection is very high, and it is accompanied by severe complications such as sepsis and migratory infection [10]. Although previous studies have shown that hvKP is in most cases susceptible to antimicrobial agents, except for ampicillin [9], hvKP has attracted growing attention due to its high pathogenicity and increasing detection rate. Indeed, once the hvKP strain becomes resistant to antibiotics, the consequences will likely be disastrous. Early reports have found a very low level of antibiotic resistance in hvKP, with less than 5% of hvKP isolates expressing extended-spectrum *β*-lactamase (ESBL) genes [12] and less than 2% demonstrating resistance to any single antibiotic tested [13]. Antibiotic-resistant hvKP strains have emerged in the past decade and caused great clinical concern. A fatal outbreak of CR-hvKP strains in China has recently been reported to pose a serious threat to public health [14]. In a 2016 study from China, more than half of hvKP isolates were shown to produce carbapenemase [15]. The spread of CR-hvKP isolates among healthy individuals may have a great global impact, given that such organisms have the potential to be the next ‘superbug’ [16]. In China, relatively high levels of hvKP and CRKP are considered the main causes of the convergence of resistance and virulence [17]. To date, an increasing number of CR-hvKP strains have been reported in areas outside China [18,21]. In general, there are two possible ways of convergence of carbapenem resistance and hypervirulence: CRKP strains obtain virulence genes or virulence plasmids, and hvKP strains acquire the chromosome or plasmid that encodes antibiotic resistance genes. Therefore, the convergence of CRKP and hvKP, with the potential for increased pathogenicity and mortality, has brought serious challenges for clinical treatment and basic research, and the transmission mechanism and epidemic characteristics need to be clarified.

An identifier of hvKP is capsule type (K). hvKP produces large amounts of capsule exopolysaccharide, which results in a hypermucoviscous phenotype (HMP) (the string test positive) when cultured on solid agar and inhibits phagocytosis, antimicrobial peptides, complement and induction of the host inflammatory response, thereby enhancing the resistance of hvKP to complement-mediated bactericidal activity and indirectly increasing its virulence [22]. Previous studies have also shown the relationships between sequence types (ST) and capsular serotypes; for example, ST23 is strongly associated with the K1 capsule type, whereas ST86 and ST23 are often associated with the K2 capsule type [23,25].

The presence of a virulence plasmid is another characteristic of hvKP strains, which is used to detect hypervirulence in a clinical setting. It has been reported that the siderophores’ iron acquisition systems and the mucoid phenotype are encoded by the plasmid and are the key determinants of the hvKP strain [26,27]. pLVPK, for example, is the well-studied virulence plasmid of hvKP strains. It is 219 kb in length and has a number of insertion sequences that are probably the result of sequential acquisition of horizontally transferred genes. It carries many virulence-associated genes, such as the capsule regulators *rmpA* and *rmpA2*, and iron acquisition genes, such as *iutA* and *iroN* [28]. There are many additional hvKP virulence factors in hvKP strains such as the ability to form biofilms and the ability to synthesize the genotoxin colibactin [29,30], but the genetic basis is unclear.

It is not appropriate to use the hypermucoviscous phenotype on the string test as the sole indicator of hvKP because not all hvKP strains are hypermucoviscous and some avirulent *K. pneumoniae* strains also have a hypermucoviscous phenotype [31,32]. Therefore, the aim of our study was to investigate the phenotype and genotype of CR-hvKP strains, reveal their resistance- and virulence-related genomic characteristics and elucidate the biological characteristics of high-virulence and high-resistance strains in order to provide molecular epidemiological data for clinical use.

## Methods

### Bacterial strains

The study included non-duplicated CRKP strains that exhibited carbapenem-resistant phenotype MIC of imipenem or ertapenem ≥4 µg ml^−1^ and were obtained from various biological samples of patients at Changhai Hospital in Shanghai, China, between January 2013 and December 2018. All of the strains were subjected to species confirmation using the Vitek two system (bioMérieux, France). Patient information was anonymously analysed and extracted from the medical records. A known hypervirulent *K. pneumoniae* isolate 1533 (hvKP1533), which carries a pLVKP-like virulence plasmid, was a gift from Dr. Yu Fy and was used as the positive control.

### String test

String test is often used to detect the hypermucoviscous phenotype of CRKP, which has been linked to hypervirulence [16]. The result was defined as positive when an inoculation loop that touched a colony pulled a ‘string’ greater than 5 mm in length.

### Antimicrobial susceptibility testing and detection of resistance genes

The MICs of 16 antibiotics were examined using the broth microdilution method, and the results were analysed in accordance with the Clinical and Laboratory Standards Institute criteria of 2019 [33]. *Escherichia coli* ATCC25922 was used as the control strain for antimicrobial susceptibility testing. Carbapenemase-encoding genes, including *bla*NDM, *bla*KPC, *bla*IMP, *bla*VIM and *bla*OXA-48, were screened by PCR amplification, as previously described [34].

### PFGE and multilocus sequence typing

PFGE with XbaI was performed for all CRKP isolates as reported previously [35]. The PFGE patterns were analysed by BioNumerics 7.6 software (Applied Maths NV, Sint-Martens-Latem, Belgium) using the dice similarity coefficient. Strains possessing ≥85% genetic similarity or fewer than four fragment differences in PFGE profiles were considered as the same clone (type). Strains (types) with patterns possessing ≥90% genetic similarity were considered to be a subtype of the outbreak strain [36].

All *K. pneumoniae* isolates were typed by multilocus sequence typing (MLST), and the sequence of seven housekeeping genes (*rpoB*, *gapA*, *mdh*, *pgi*, *phoE*, *infB* and *tonB*) was amplified and analysed from the Pasteur Institute *K. pneumoniae* MLST database website (http://bigsdb.pasteur.fr/klebsiella/klebsiella.html) [37].

### Capsular polysaccharide synthesis genotyping

Multiplex PCR analysis was designed as previously reported [38] to detect the capsular polymerase genes K1, K2, K47 and K64 – the most prevalent capsular types of ST11 strains in China [39].

### Virulence characteristics: infection of *Galleria mellonella* larvae

We tested the virulence potential of the isolates in *G. mellonella* larvae (wax worms) weighing ~300 mg (purchased from Huiyude Biotech Company, Tianjin, China), as previously described [40]. Control experiments were performed with *K. pneumoniae* ATCC700603.

### Genome sequencing, virulence genes, antimicrobial resistance genes and analysis of plasmids

Representative strains were further analysed by next-generation sequencing (NGS) to better understand the genetic basis of CR-hvKP strains. The genomes of the isolates were subjected to sequencing using 150 bp reads on an Illumina HiSeq 2500 sequencer in accordance with the manufacturer’s instructions, at ~100× coverage. Genomic sequence contigs were *de novo* assembled using the MicrobeTrakr Plus (Zeta Biosciences, Shanghai, China). In accordance with the manufacturer’s instructions, Quake and BWA were used in pre- and post-assembly sequence corrections, respectively [41].

We identified the antibiotic resistance genes and virulence loci with the assembled genome sequences using ResFinder3.2 [42]. The virulence genes of *K. pneumoniae* were detected from the VFDB [43] and VRprofile 2.0 (http://202.120.12.134/STEP/STEP_VR.html).

The genome structure comparisons were performed to analyse the sequence homology with pLVPK virulence-associated plasmids. The blast Ring Image Generator was used to compare the plasmids with the highly homologous plasmids in the NCBI database and display a plasmid circular structure map [44].

## Results

A retrospective review of all cases of CRKP diagnosed at our microbiological laboratories between January 2013 and December 2018 identified 500 cases of CRKP. Of the 500 non-duplicated CRKP isolates, 18(3.6%) were positive on the string test and were identified as CR-hvKPs. Among them, 12 were found in men and 6 were found in women. The average age of the patients was 63 years old, ranging from 47 to 95 years old. The patients were from nine wards, including the emergency intensive care unit (ICU) (eight cases), emergency observation ward (two cases), cardiovascular ICU (two cases), burn ICU (one case) and other five wards (one case each). Ten isolates were collected from sputum samples, four isolates from blood samples, two isolates from urine samples, one isolate from an abdominal secretion sample and one isolate from a bile sample.

Sixteen antibiotics were used for antimicrobial susceptibility testing of the 18 CR-hvKPs. All of the 18 isolates exhibited resistance to most of the tested antimicrobial agents, including ertapenem or imipenem. The results of the antimicrobial susceptibility testing are summarized in Table 1 (not show all). Our results indicated that all these isolates were multidrug-resistant, exhibiting high-level resistance to most *β*-lactam antibiotics, including carbapenems, but some isolates remained susceptible to amikacin.

**Table 1.** Resistance genes, virulence genes and antibiotic resistance characteristics of the 18 CR-hvKP strains

**No.**	**MLST**	**Capsular serotype**	**PFGE**	**Resistant gene**			**Virulence gene**	**Minimum inhibitory concentration**										
				**Carbapenems**	**β-lactamase**	**Quinolone**		**SAM**	**TZP**	**CZO**	**CAZ**	**CRO**	**FEP**	**ETP**	**IPM**	**AMK**	**CIP**	**LE**
4,6	ST11	K64	A1	KPC-2	*blaDHA-1, blaSHV-182, blaTEM-1B*	*oqxAB,qnrB4*	*entB, fimH, mrkD, ybtS*	≥32	≥128	≥64	≥64	≥64	≥64	≥8	8	≥64	≥4	≥8
5	ST1412	-	H	-	*blaCTX-M-14, blaOXA-1,blaSHV-145, blaTEM*	*oqxAB, qnrB52, qnrS1*	*rmpA,rmpA2, iroN, iutA, iucA,entB, fimH, iroB, iroC, iroD, mrkD, ybtS*	≥32	64	≥64	4	≥64	≥64	4	≤1	≤2	≥4	1
9	ST12	K64	A2	KPC-2	*blaCTX-M-123, blaCTX-M 65,blaSHV-182, blaTEM-1B*	-	*entB, fim, mrkD, ybtS*	≥32	≥128	≥64	≥64	≥64	≥64	≥8	≥16	≥64	≥4	≥8
11	ST65	K2	I	KPC-2	*bla*CTX-M-15,*bla*OXA-1,*bla*SHV-11,*bla*TEM-1B	*oqxAB,qnrS1*	*rmpA,rmpA2,iroN,iutA,iucA,entB,fimH,iroB,iroC,iroD,mrkD,ybtS*	≥32	≥128	≥64	≥64	≥64	8	≥8	8	≤2	≥4	≥8
10,12	ST11	K64	C	KPC-2	*bla*CTX-M-65,*bla*TEM-1B,*bla*SHV-12	*qnrS1*	*rmpA,rmpA2,iroN,iutA,iucA,entB,fimH,mrkD,ybtS*	≥32	≥128	≥64	≥64	≥64	≥64	≥8	≥16	≥64	≥4	≥8
13	ST268	–	J	KPC-2	*bla*SHV-11	*oqxAB*	*rmpA,rmpA2,iroN,iutA,iucA,entB,fimH,iroB,iroC,iroD,mrkD,ybtS*	≥32	≥128	≥64	8	16	2	4	≤1	≤2	≤0.25	≤0.25
14,15,16,17	ST11	K64	F	KPC-2	*bla*SHV-12	*qnrS1*	*entB,fimH,mrkD,ybtS*	≥32	≥128	≥64	≥64	≥64	≥64	≥8	≥16	≤2	≥4	≥8
19	ST11	K64	D	KPC-2	*bla*CTX,M-65,*bla*SHV-182	–	*rmpA,rmpA2,iutA,iucA,entB,fimH,mrkD,ybtS*	≥32	≥128	≥64	≥64	≥64	≥64	≥8	≥16	≤2	≥4	≥8
20	ST11	K47	B	KPC-2	*bla*CTX-M-65,*bla*SHV-12	*oqxAB*	*entB,fimH,mrkD,ybtS*	≥32	≥128	≥64	≥64	≥64	≥64	≥8	≥16	≥64	≥4	≥8
21	ST11	K64	E2	KPC-2	blaCTX-M-65,*bla*TEM-1B,*bla*SHV-12	*qnrS1*	*rmpA,rmpA2,iroN,iutA,iucA,entB,fimH,mrkD,ybtS*	≥32	≥128	≥64	≥64	≥64	≥64	≥8	≥16	≥64	≥4	≥8
22	ST11	K64	G	KPC-2	*bla*CTX-M-65	*qnrS1*	*rmpA,rmpA2,iutA,iucA,entB,fimH,mrkD,ybtS*	≥32	≥128	≥64	≥64	≥64	≥64	≥8	≥16	≥64	≥4	≥8
23,24	ST11	K64	E1	KPC-2	*bla*CTX-M-14,*bla*SHV-12	*qnrS1*	*rmpA,rmpA2,iroN,iutA,iucA,entB,fimH,mrkD,ybtS*	≥32	≥128	≥64	≥64	≥64	≥64	≥8	≥16	≤2	≥4	≥8

AMKamikacinCAZceftazidimeCIPciprofloxacinCROceftriaxoneCZOcefazolinETPertapenemFEPCefepimeIPMimipenemLVXlevofloxacinSAMampicillin/sulbactamTZPpiperacillin/tazobactam

Carbapenemase genes, including *bla*NDM, *bla*KPC, *bla*IMP, *bla*VIM and *bla*OXA-48, were rapidly confirmed by multiplex PCR analysis in all 18 strains. Seventeen were positive for *bla*KPC-2, while none of the carbapenemase genes were found in one isolate (N5) with a carbapenem-resistance phenotype. Another multiplex PCR analysis was used to detect the capsular polymerase gene, including K1, K2, K47 and K64. Among the 18 CR-hvKP isolates, 14 (77.8%) belonged to capsular serotype K64, 1 (5.6%) belonged to K2, and another 1 (5.6%) belonged to K47. The remaining two isolates (11.0%) were not successfully typed and were therefore defined as K-nontypable. The results are summarized in Table 1.

**Fig. 1.**
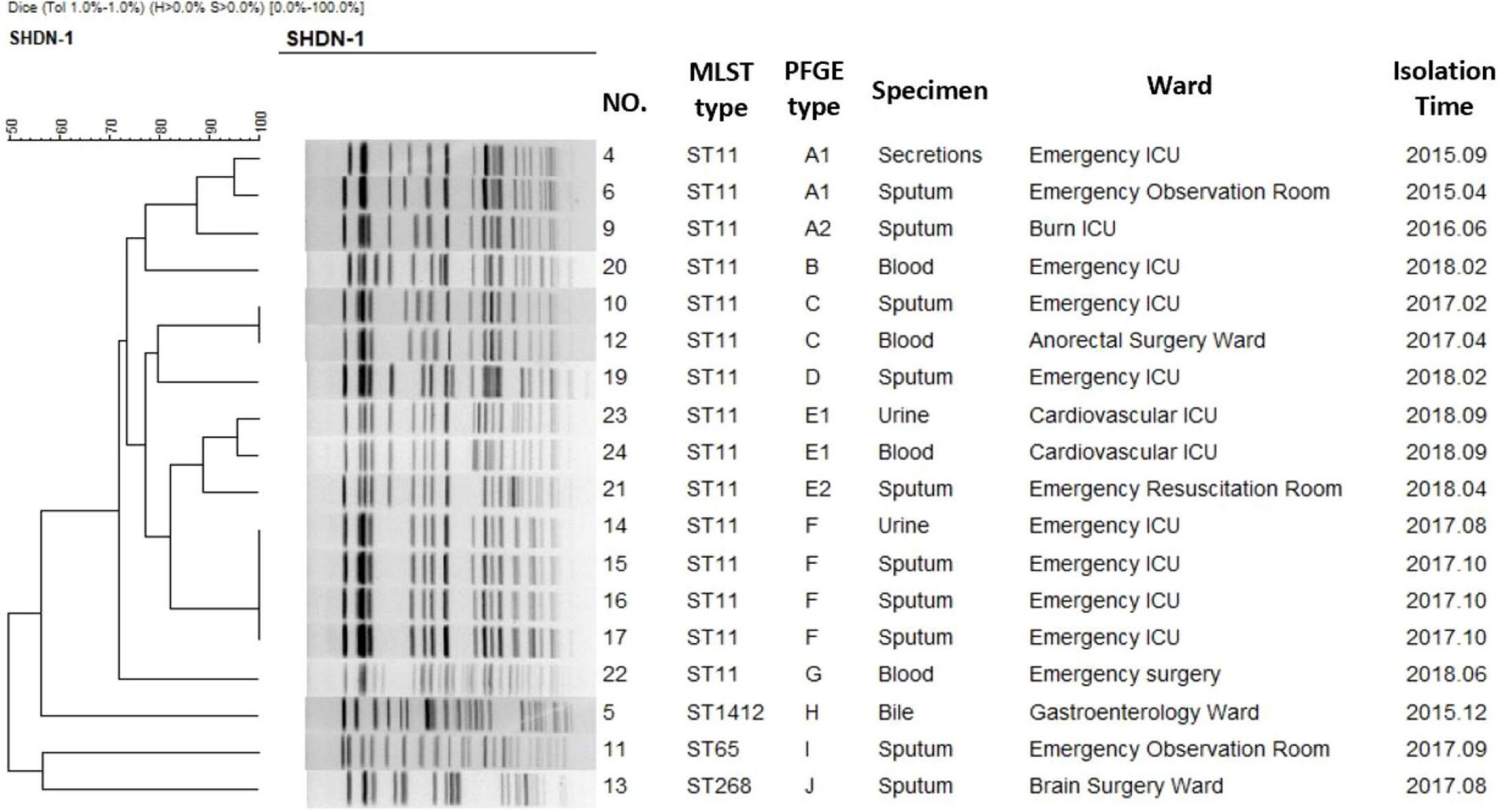
Homology of 18 CR-hvKP isolates.

To assess the genotypes of the CR-hvKP isolates, MLST was performed. Among the 18 CR-HMKP isolates, 15 (83.3%) belonged to the ST11 group. The remaining three isolates belonged to ST1412, ST65 and ST268. PFGE analysis identified ten unrelated PFGE types (A–J), with four PFGE subtypes within these types (A1, A2, E1 and E2) (Fig. 1)

**Fig. 2.**
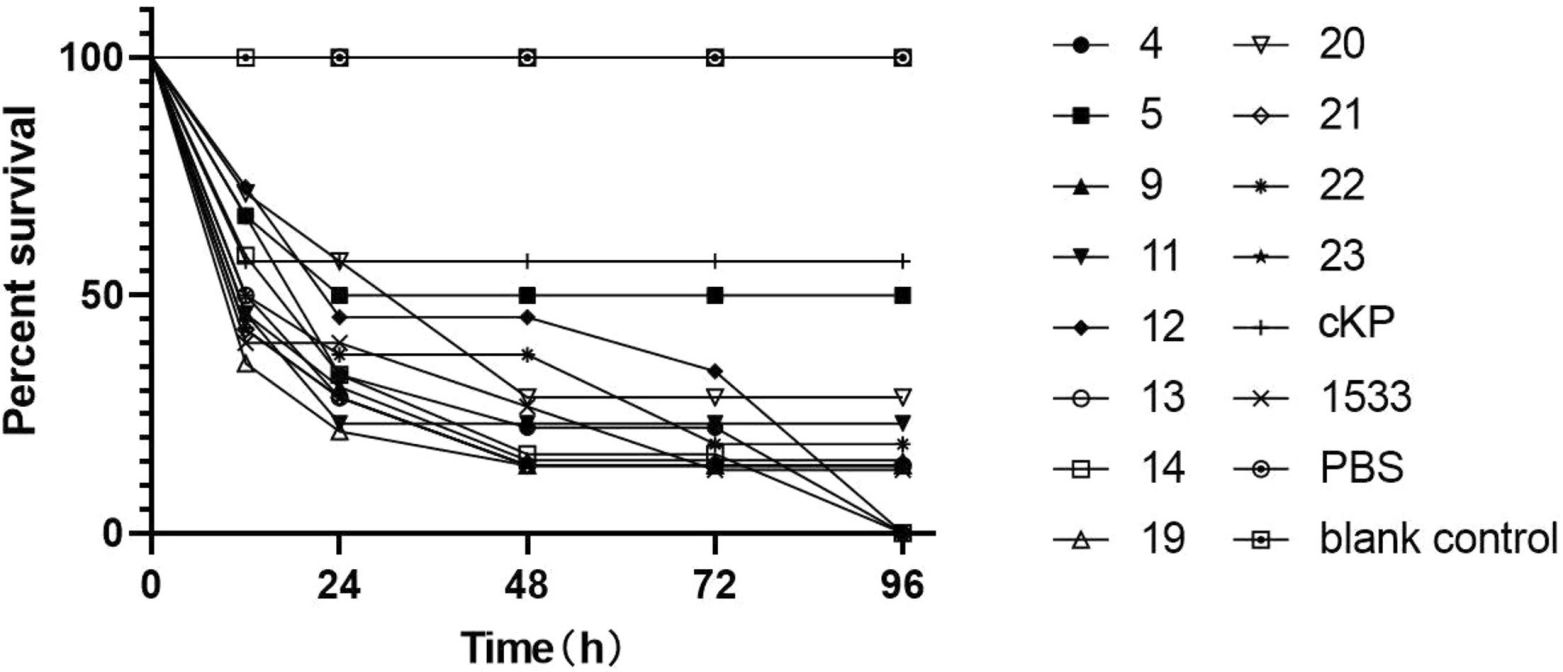
Survival of *Galleria mellonella* larvae infected with 12 representative CR-hvKP isolates. A hypervirulent *K. pneumoniae* K2 strain hvKP1533 was used as the positive control. A PBS-injected, a pricking larval group (empty needle injection, blank control) and cKP served as negative control groups.

Based on the results of PFGE, we selected one strain from each PFGE type for virulence-associated phenotype experiments. A total of 12 CR-hvKP isolates of different types and subtypes were selected. Further animal testing results using a *G. mellonella* infection model are shown in Fig. 2. Specifically, we infected *G. mellonella* larvae with the selected *K. pneumoniae* isolates (inoculum of 1×10⁶ c.f.u.). The results showed that after 48 h of infection, the larvae in the CR-hvKP groups had lower viability than the classical *K. pneumoniae* (cKP) group, phosphate-buffered saline (PBS) group and blank control. Generally, all of these isolates showed relatively higher virulence than cKP and similar to that of hvKP1533, which is known to carry a pLVPK virulence plasmid.

**Fig. 3.**
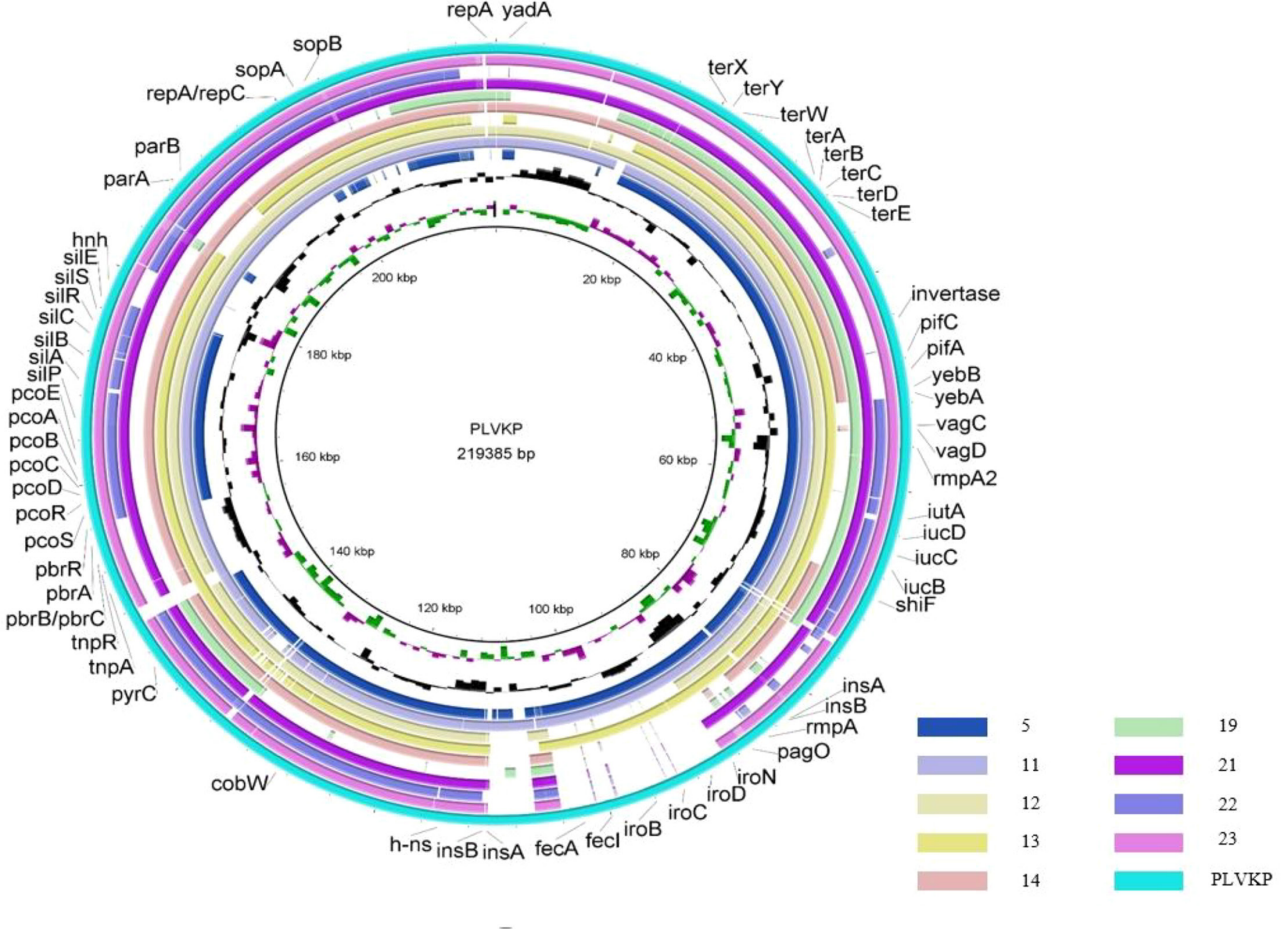
Comparative genomic circles of nine representative CR-hvKP isolates. Among the 12 assembled genomes, 3 isolates were eliminated due to insufficient matching data with the pLVPK plasmid.

NGS was used to understand the genetic basis of these 12 CR-hvKP isolates, and we compared the results with the antibiotic resistance gene database. Comparative genomic analysis of the antibiotic-resistance genes indicated that the CR-hvKP strains contained many types and many drug-resistance genes. These CR-hvKP isolates carried almost all classes of genes conferring resistance to sulphonamide, tetracycline, trimethoprim, beta-lactam, rifampicin, aminoglycoside, fosfomycin, quinolone, macrolide and phenicol (Fig. 3). In addition to *bla*KPC-2, 6 of these 12 isolates carried extended-spectrum *β*-lactamase gene *bla*CTX-M-65, while 7 isolates carried *bla*TEM-1B. Virulence genes of CR-hvKP isolates were detected from the Virulence Factor Database (VFDB) (Fig. 3). The virulence-associated genes with 100% detection rates among the 12 isolates included *entB* (100%, 12/12), *fimH* (100%, 12/12), *mrkD* (100%, 12/12) and *ybtS* (100%, 12/12), and other genes included *rmpA* (67%, 8/12), *rmpA2* (67%, 8/12), *iutA* (67%, 8/12), *iucA* (67%, 8/12), *iroN* (50%, 6/12) and *iroBCD* (25%, 3/12).

We compared the genotypes of the CR-hvKP isolates with pLVPK virulence-associated plasmids. Among the 12 assembled genomes, 3 isolates were assembled with little data and then eliminated. The results showed that the remaining nine isolates had homology with pLVPK (Fig. 3). Alignment of contigs suggested that 9 of the 12 ST11 CR-hvKP isolates carried a plasmid that aligned well to many parts of the pLVPK plasmid, except N14 isolates; others included the region in which the *rmpA*, *rmpA2*, *iucABCD* (aerobactin) and *iutA* (aerobactin receptor) genes were located. N5, N11 and N13 had *iroBCDN* (salmochelin) genes, and N12, N21 and N23 had only *iroN*. The results are in line with previous studies from the VFDB database.

## Discussion

In this study, we described the epidemiology of CR-hvKP in patients in a teaching hospital in Shanghai, China, from 2013 to 2018, and the MLST assay revealed that most CRKP strains were ST11. We also described the genetic basis of drug resistance and virulence of these strains.

Our results revealed the emergence of ST11 CR-hvKP strains in our hospital, which requires special attention. After the acquisition of a virulence plasmid by classic ST11 CRKP strains, these strains are now both hypervirulent and multidrug-resistant and could therefore be regarded as a real ‘superbug’ that could represent a serious risk to public health. Earlier studies have shown that hvKP strains are sensitive to most antibiotics, whereas multidrug-resistant *K. pneumoniae* strains have lower virulence, and they have evolved separately in different clonal groups [14,45]. However, we screened 18 CR-hvKP isolates out of 500 strains of CRKP and showed that a high-virulence phenotype also contained almost all types of antibiotic-resistance genes. Although many ST11 CR-hvKPs have been reported in China, ST11-K64 has rarely been reported. A previous study reported an ST11-K64 strain, which was isolated from a patient with pyogenic liver abscess [46]. The strains included in our study came from eight clinical departments from different patients with different diseases, and the samples were taken from sputum, blood, urine and abdominal secretions. As PFGE showed that these strains originated from ten unrelated PFGE types, they were sporadic and spread unsteadily. Among these strains, N14, N15, N16 and N17 belonged to the same PFGE group; they occurred within 2 months, and all of them were found in the emergency ICU ward, representing a small outbreak of CR-hvKPs.

According to the gene-sequencing results, we further compared the antimicrobial resistance genes and virulence genes with the database. Consistent with the previous report [46], compared with ST11-K64 CR-hvKPs, ST11-K47 CR-hvKPs lacked traditional virulence genes such as *rmpA/rmpA2* genes and siderophore gene clusters such as *iucABCD*, *iroBCDN* and *iutA*. In addition, not all ST11-K64 strains contained these virulence genes, for example, N4 and N9 ST11-K64 CR-hvKPs lacked *rmpA/rmpA2* genes and siderophore genes.

Comparative genomic analysis of the antibiotic-resistance genes indicated that both ST11-K64 and ST11-K47 strains contained many types and many drug-resistance genes. N11 was an ST65 strain, which primarily belongs to hypervirulent capsular K2 strains [16]. Interestingly, there was one ST1412 strain with a phenotype resistant to carbapenems, but without common carbapenem-resistance genes detected. Hence, it is critical to note that the production of carbapenemases is not the only mechanism of acquired resistance to carbapenems, and there could be other mechanisms such as alterations in outer membrane permeability mediated by the loss of outer membrane proteins.

In this study, we observed that CR-hvKP strains exhibited a trend of pan-drug resistance, resulting in difficulties in clinical treatment. Plazomicin, eravacycline, cefiderocol, temocillin, ceftolozane–tazobactam, imipenem–cilastatin/relebactam, meropenem–vaborbactam, ceftazidime–avibactam and aztreonam–avibactam constitute potent alternatives for treating CRKP infections [47]. However, monotherapy for CRKP infections is less effective compared with combination therapy [48]. The current recommended combination therapy for CRKP is based on polymyxins, tigecycline or ceftazidime–avibactam, in combination with aminoglycosides, colistin, carbapenems, fluoroquinolones and fosfomycin for anti-infective treatment.

Most of the CR-hvKPs strains in this study had homology with a pLVKP virulence plasmid – probably due to the acquisition of a virulence plasmid by classic ST11 CRKP strains – which resulted in simultaneous hypervirulent and multidrug-resistant phenotype. The study is limited by the small number of strains and the lack of detailed case tracking, making it impossible to determine clinical manifestations of highly virulent strain infections.

## supplementary material

10.1099/jmm.0.001960 Supplementary Material 1.

## Abbreviations

CR-hvKP: carbapenem-resistant hypervirulent *Klebsiella pneumoniae*
CRKP: carbapenem-resistant *Klebsiella pneumoniae*
ESBL: extended-spectrum *β*-lactamase
hvKP: hypervirulent *Klebsiella pneumoniae*
ICU: intensive care unit
KPC: *Klebsiella pneumoniae* carbapenemase
MLST: multilocus sequence typing
NGS: next-generation sequencing
ST11: sequence type 11
VFDB: virulence factor database
