# Supplementary material for: Phenotype and genotype of carbapenem-resistant hypervirulent Klebsiella pneumoniae in a teaching hospital in Shanghai, China
Source: J Med Microbiol. 2025 Mar 5;74(3):001960. doi: 10.1099/jmm.0.001960 (PMC11897171; doi:10.1099/jmm.0.001960)
Supplement: Supplementary Material 1. [file jmm-74-01960-s001.pdf]

Sequence data that support the findings of this study have been deposited in the National Center for Biotechnology Information.

PRJNA1067379	Klebsiella pneumoniae hmvpk_5
PRJNA1067390	Klebsiella pneumoniae hmvpk_9
PRJNA1067402	Klebsiella pneumoniae hmvpk_11
PRJNA1067421	Klebsiella pneumoniae hmvpk_13
PRJNA1067424	Klebsiella pneumoniae hmvpk_14
PRJNA1067737	Klebsiella pneumoniae hmvpk_19
PRJNA1067752	Klebsiella pneumoniae hmvpk_20
PRJNA1067808	Klebsiella pneumoniae hmvpk_21
PRJNA1104409	Klebsiella pneumoniae hmvpk_22
PRJNA1067815	Klebsiella pneumoniae hmvpk_23
